# Supplementary figures and images for: Patient delay and associated factors among tuberculosis patients in Gamo zone public health facilities, Southern Ethiopia: An institution-based cross-sectional study
Source: PLoS One. 2021 Jul 30;16(7):e0255327. doi: 10.1371/journal.pone.0255327 (PMC8323940; doi:10.1371/journal.pone.0255327)

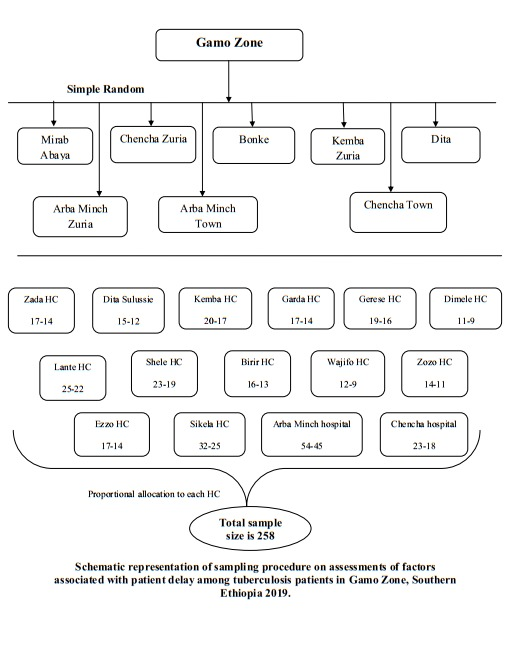

Supplement: S1 Fig — (TIF) [file pone.0255327.s001.tif]
