# Supplementary material for: Patient delay and associated factors among tuberculosis patients in Gamo zone public health facilities, Southern Ethiopia: An institution-based cross-sectional study
Source: PLoS One. 2021 Jul 30;16(7):e0255327. doi: 10.1371/journal.pone.0255327 (PMC8323940; doi:10.1371/journal.pone.0255327)
Supplement: S1 Text — (DOCX) [file pone.0255327.s002.docx]

**Operational definition and definition of terms**

Different delay periods were defined similarly to previous studies, and median value was used as a cut-off value to make simple comparison with previous similar studies.

**Patient delay**

The time interval (in days) between the initial onsets of the first symptoms of TB until the first visit to a formal health care provider. TB patients who consulted a formal health care provider longer than the median value after the onset of the initial constitutional signs and symptoms of TB were considered delayed[1][2].

**Onset of Tuberculosis symptom**

The time at which the first symptom (i.e. Cough and other constitutional symptoms like fever, weakness, and weight loss or chest pain) of the illness for which a patient’s health care seeking began[3].

**Formal-health care providers:** modern government or private health care facilities such as clinics, health centers and hospitals[2].

**Non-formal health providers:** These include traditional health providers, local injectors and drug retail outlets[2].

**The different category of TB patients is based on the national TB control guideline**[4]**, and operationalized as follow:**

***Pulmonary tuberculosis (PTB)*** refers to any bacteriologically confirmed or clinically diagnosed case of TB involving the lung parenchyma or the tracheobronchial tree.

***Smear positive PTB*** refers to a patient with at least one sputum smear positive for acid-fast bacilli (AFB), or one sputum smear positive for AFB plus radiographic abnormalities consistent with active pulmonary tuberculosis; or one sputum specimen positive for AFB plus culture specimen positive for Mycobacterium tuberculosis.

***Smear-negative PTB*** refers to a patient with two negative sputum smears for AFB and radiological abnormality consistent with active TB or failure to respond to antibiotics treatment or one which health worker or clinician has diagnosed TB and decided to treat the patient with full course of anti TB drugs.

***Extra pulmonary tuberculosis (EPTB)*** refers to any bacteriologically confirmed or clinically diagnosed case of TB involving organs other than the lungs, e.g. pleura, lymph nodes, abdomen, genitourinary tract, skin, joints and bones, meninges.

**Multiple Healthcare contacts** defined as making more than one visit to any public health facility irrespective of their type as government or private before TB diagnosis confirmation is made[2].

**TB-Associated Stigma:** Variables measuring stigma was recorded on a 5-point Likert scale of nine questions (five the highest and one the lowest degree of stigma). These variables included feeling ashamed of having tuberculosis; having to hide tuberculosis diagnosis from others; delay to seek treatment due to fear of being diagnosed with TB; isolation due to tuberculosis; tuberculosis affecting the relationship with others; fear of TB/HIV co infection. Patients who scored more than set average (50%) will be considered having a high TB-associated stigma and those who scored less than average will be considered having a low TB-associated stigma[5].

**Knowledge about TB:** was assessed using eight items with “yes” or “no” questions including cause of TB (microbe, bacteria, germ), TB is hereditary, TB is contagious, mode of TB transmission (breathing, sneezing, coughing, raw milk intake), symptoms of TB, TB is curable, length of treatment (6 month=yes, otherwise no) and TB treatment modalities as free=yes or for charge=no. Patients who scored more than set average (50%) was considered knowledgeable and those who scored less than average were considered not knowledgeable[5].

**Travel time to health facility:** The time it tooks to reach the nearest health facility at the time of illness from place of residence. In this study, patient will be considerd from long distance if the time took to reach health facility morthan 30 minutes[2].

**The body mass index (BMI) in kg/m^2^** will be computed to determine the nutritional status of the patients at date of presentation to health facility will be classified as underweight <18.50, normal range 18.50-24.99, overweight >25.00-29.99 and obese over 30[6].

**Food Insecurity Experience Scale**

FIES is comprised of eight questions ranging in the severity of FI (Food insecurity) they measure, from low FI (question 1) to Severe Food insecurity (question 8). Respondents answer yes/no to the 8 questions and the responses are aggregated to give raw scores ranging from 0 to 8. FI was classified into 3 categories: 1) food secure (FS) with raw scores = 0–3; 2) moderate FI (MFI), with raw scores = 4–6; and 3) Sever FI, with raw scores = 7–8[7][8].

**References**

1. Kalan ME, Sis HY, Kelkar V, Harrison SH, Goins GD, Jafarabadi MA, et al. The identification of risk factors associated with patient and healthcare system delays in the treatment of tuberculosis in Tabriz, Iran. BMC Public Health. 2018;18(1):174.

2. Seid A, Metaferia Y. Factors associated with treatment delay among newly diagnosed tuberculosis patients in Dessie city and surroundings, Northern Central Ethiopia: a cross-sectional study. BMC Public Health. 2018;18(1):931.

3. Lusignani LS, Quaglio G, Atzori A, Nsuka J, Grainger R, Palma MDC, et al. Factors associated with patient and health care system delay in diagnosis for tuberculosis in the province of Luanda, Angola. BMC Infect Dis. 2013;13(1):168.

4. Ethiopia FM of H of. GUIDELINES FOR CLINICAL AND PROGRAMMATIC MANAGEMENT OF TB, TB/HIV AND LEPROSY IN ETHIOPIA . ADDIS ABABA : Federal Minstry of Health of Ethiopia; 2016.

5. World Health Organization (WHO) and Regional Ofice for the Eastern Mediterranean. Diagnostic and Treatment Delay in Tuberculosis. An InDepth Analysis of the Health-Seeking Behaviour of Patients and Health System Response in Seven Countries of the Eastern Mediterranean Region. [Internet]. Geneva, Switzerland: World Health Organization (WHO); 2006. Available from: http://applications.emro.who.int/dsaf/dsa710.pdf

6. Organization WH. Obesity: preventing and managing the global epidemic: report of a WHO consultation on obesity, Geneva, 3-5 June 1997. World Health Organization; 1998.

7. FAO. The state of food insecurity in the world 2015. Meeting the 2015 international hunger targets: taking stock of uneven progress [Internet]. Rome: Food and Agriculture Organization of the United Nations, International Fund for Agricultural Development, World Food Programme; 2015. Available from: http://www.fao.org/3/a-i4646e.pdf.

8. Ballard TJ Cafiero C, KAW. The food insecurity experience scale: developing a global standard for monitoring hunger worldwide. [Internet]. Rome: FAO; 2013. p. Technical paper [Internet]. Available from: http://www.fao.org/fileadmin/templates/ess/voh/FIES_Technical_Paper_v1.1.pdf.
