# Supplementary material for: Patient delay and associated factors among tuberculosis patients in Gamo zone public health facilities, Southern Ethiopia: An institution-based cross-sectional study
Source: PLoS One. 2021 Jul 30;16(7):e0255327. doi: 10.1371/journal.pone.0255327 (PMC8323940; doi:10.1371/journal.pone.0255327)
Supplement: S1 File — (PDF) [file pone.0255327.s003.pdf]

Annex 3: English version questioners

Questionnaire ID________________ Name of the Woreda

Type of Health facility

1. Hospital 2. Health center

Name of the health facility______________________ Date of interview

Name of interviewer______________________________Signature

Name of supervisor_______________________________ Signature

**Unit TB register review**		**Remark**
TB Unit number		
Sex	1. Male 2.Female	
Age	_______________Years	
Smear Results	1. Positive<br>2. Negative<br>3. Not done	
Nutritional status of the patients	Baseline/initial weight_________________(Kg)<br><br>Height_________________(90)	
Patient category	1. New<br>2. Transfer in	
Type of TB	1. SPPTB<br>2. SNPTB<br>3. EPTB	
Treatment started	______________________ date/dd/mm/yy	
HIV result	1. Reactive<br>2. Non-reactive<br>3. Unknown	

PART I: PATIENTS' GENERAL BACKGROUND INFORMATION (SOCIO DEMOGRAPHIC AND SOCIOECONOMIC CHARACTERISTICS)

Q no	Questions	Options/responses	Skip
101	Your usual residence	1. Urban<br>2. Rural	
102	Your current marital status	1. Never married<br>2. Married<br>3. Widowed<br>4. Divorced/Separated	
103	To which religion do you belong?	1. Orthodox Christian<br>2. Muslim<br>3. Catholic<br>4. Protestant Christian<br>5. Traditional<br>6. Other specify--------------	
104	What is your highest level of education Completed?	1.Never attended formal school/Illiterate<br>2. Primary school(1-8)<br>3. Secondary school(9-12)<br>4. Collage and above<br>5. Other (specify--------------	
105	What is your main occupation(prior to the disease)?	1. Government employee<br>2. Private employee<br>3. Self employed<br>4. Housemaid<br>5. House wife<br>6. Student<br>7. Farmer<br>8. Daily laborer<br>9. Merchant<br>10. Other (specify--------------	
106	Your family size/ household members	______________in number	
107	Number of rooms in the households?	_______________in number	
108	Monthly income	_______________in Birr	
109	Are you a member of community-based health insurance?	1. Yes<br>2.No	

PART II. CLINICAL CHARACTERISTICS AND HEALTH CARE SEEKING BEHAVIOR

201	When was the current illness you are being treated started?	1. -------/---------/-------dd/mm/yy<br>2. I don't know/remember
202	What was/were the first symptom(s) made you seek care?<br>***Note for interviewer:*** *More than one response possible and check for that without reading options*	1.Cough<br>2. Fever<br>3. Loss of Weight<br>4. Hemoptysis<br>5. Chest pain<br>6. Breathlessness/Dyspenia<br>7. Night sweating<br>8. Fatigue/Weaknes<br>9. Loss of appetite<br>10. Others (specify-----
203	Where did you first seek care for the complaints?	1. Self-medication<br>2. Used traditional medicine<br>3. Used holly water<br>4. Consult HEW<br>5. Consult HCP at HF<br>6. Other (specify--------------)
204	Reasons of first consultation of the health facility (mentioned in q.204) with the onset of symptoms (i.e 5 and 6 in q.204) (check):	1. Accessible<br>2. Confidence in getting cured<br>3. Services available anytime<br>4. Free services<br>5. Advised by somebody<br>6. Others (specify)
205	Why did not you consult health facility first during the onset of symptoms?<br>***Note for interviewer:*** *Health facility is to mean private clinic or hospital, public health center, or hospital or health post*<br>*More than one response possible and check for that without reading options*	1. Thought mild and relived by it self<br>2. Health facilities were too far<br>3. Facility do not provide the service<br>4. Too busy/long waiting time<br>5. Previous bad experiences<br>6. Fear of being diagnosed for TB<br>7. TB is common and cure by itself<br>8. Fear of HIV test<br>9. I don't know service is provided there<br>10. I thought the cost is too expensive<br>11. Mistrust of health services provision<br>12. My belief system don't allow<br>13. Other (specify)-----------------------
206	Which HCF did you first visit?	1. Health post<br>2. Public Hospital<br>3. Health center<br>4. Private (Hospital/Clinic)<br>5. Others (specify)----------------------
207	What was your functional status during presentation to HCF?	1. Working<br>2. Ambulatory<br>3. Bed ridden

208	How far is the HCF you visited first from your usual residence?	____________minute/hour
209	In total how long time, did you spend from the onset of illness to the first HCF visit?	_________________days/weeks/months
210	Do you think that you delayed consultation of HCP for your illness?	1. Yes, if yes, go to question no **211.**<br>2. No, if no, go to question no.**212.**
211	If felt delayed, Why did you delay consultation of HCP at health facilities?<br>*Possible perceived cause of delay*	1. Hoped symptoms would go away by themselves<br>2. Fear of social isolation<br>3. Fear of being diagnosed for TB/ Fear of what would be found in diagnosis<br>4. Fear of routine HIV test at health facilities<br>5. Lack of money to cover consultation fees<br>6. Bad staff attitude to patients<br>7. Perceived poor qualities of health services<br>8.Busy occupational life intervened in consulting HCF<br>9.Transport and long distance<br>10.Tought like common illness<br>8. Others (mention-------------------------)
212	When was your final diagnosis of TB made?	---------------------------------dd/mm/yyyy<br>2. I don't know/remember
213	Where was the final diagnosis of TB made?	1. Health center<br>2. Public hospital<br>3. Private institution<br>4. Other
214	Until the final diagnosis of TB, how many ***facilities*** did you visit?	_____________in number
215	How many ***visits*** did you made to HCF until the final diagnosis of TB?	_____________in number
216	How long time did it take since you first visited HCF until you were informed you had TB?	_________________days/weeks/months
217	Do you think that diagnosis of TB was delayed after you made visits to HF?	1. Yes, if yes, go to question no. **218.**<br>2. No, if no, go to question no.**219.**
218	If felt delayed, what do you think is/are the reasons for the delay?<br>***Note for interviewer:*** *More than one response possible and check for that without reading options*	1.Failure of providers to reach at diagnosis<br>2.Lack of facilities/supplies for diagnoses<br>3. Prescription of unnecessary drugs<br>4.Repeated referrals to different facilities<br>5. Other (specify)-----------------------

219	Types of diagnostic test used	1.Microscope<br>2. Chest X-ray<br>3.Microscope and Chest X-ray<br>4. Others
220	Do you have history of TB contact in the last one year?	1. Yes ⟶ go to question no 227.<br>2. No
226	Point of contact	1. House holds<br>2. In the facility<br>3. College<br>4. At work<br>5. School<br>6. Others

PART III: ANTI-TB TREATMENT PRACTICES

Q no	Question	Options /response
301	When did you start anti-TB treatment?	______________________dd/mm/yy<br>2. I don't know/remember
302	How long it takes you to start TB treatment since first HCP consultation.	--------------------days/weeks/months
303	How long after the diagnosis of TB that you commenced anti-TB treatment?	1. Immediately<br>2. ----------------days/weeks(304)
304	Why you did not start treatment immediately?	1. I was reluctant to initiate the treatment<br>2. Fear of long treatment<br>3. Lack of anti-TB drugs at the facility<br>4. TB clinic was closed<br>5. Absence of DOT provider<br>6. Failure to present treatment supporter<br>7. Inability to arrange accommodation at nearby<br>8. Too ill to initiate early<br>9. Other (specify-----------------------

PART IV: KNOWLEDGE AND PERCEIVED STIGMA RELATED TO TUBERCULOSIS

**Patient's knowledge on TB**

401	What kind of disease are you being treated currently?	1. TB<br>2. Pneumonia<br>3. Cold<br>4. I donot know<br>5. Other (specify)-------------------
402	Have you ever heard of TB illness before?	1. Yes<br>2. No———▶403
403	Where did you hear about TB (source of information on TB)<br>***Note for interviewer****: More than one response possible and check for that without reading option*	1.Mass media (radio,TV,news letter,etc)<br>2.MOH banners,brochers,tamphlet etc<br>3. HEW<br>4. Health facility<br>5. Friends/relatives<br>6. TB case treated/on treatment<br>7. Other (specify)------------------
404	What do you think is the causative agent of TB?	1. Bacteria<br>2. Smoking cigarette<br>3. Exposure to cold<br>4. Fungus<br>5. Virus<br>6. Curse<br>7. Other (Specify)------------------<br>8. I don't know
405	Is TB contagious?	1. Yes if yes, go to question no **406.**<br>2. No, if no, go to question no.**407.**
406	If contagious, how can a person get tuberculosis (TB transmission)?<br>***Note for interviewer:*** *More than one response possible and check for that without reading options*	1. Through the air when coughing or sneezing<br>2. Through sharing utensils<br>3. Through touching a person<br>4. Through food<br>5. Through sexual contact<br>6. Through mosquito bites<br>7. Through drinking unboiled milk<br>8. Other (specify)-------------------<br>9. I Don't know
407	What symptoms does a person with tuberculosis have?<br>***Note for interviewer****: More than one response possible and check for that without reading options*	1.Persistent cough(greater than 2weeks)<br>2. Coughing up blood<br>3. Weight loss<br>4. Poor appetite<br>5. Night sweating<br>6. Chest pain<br>7. Fever<br>8. Other (specify)-------------------<br>9. Don't know
408	Is TB hereditary?	1. Yes<br>2. No
409	Do you know if there is a vaccine for TB?	1. Yes<br>2. No
410	How is treatment of TB provided?	1. For free<br>2. For charge
411	Do you know the approximated duration of TB treatment?	1. Yes (specify-----------<br>2. No

412	Is tuberculosis curable?	1. Yes<br>2. No

Perceived Stigma Related Question		
414	Do you feel ashamed for having this disease?	1. Strongly agree<br>2. Agree<br>3. Not sure<br>4. Disagree<br>5. Strongly disagree
415	Do you have to hide other people that you have diagnosed that you have this disease?	1. Strongly agree<br>2. Agree<br>3. Not sure<br>4. Disagree<br>5. Strongly disagree
416	Does this disease affect relation with the others?	1. Strongly agree<br>2. Agree<br>3. Not sure<br>4. Disagree<br>5. Strongly disagree
417	Is the disease very costly to you due to its long duration of treatment?	1. Strongly agree<br>2. Agree<br>3.Not sure<br>4. Disagree<br>5. Strongly disagree
418	Do you prefer to live isolated since you diagnosed that you have this disease?	1. Strongly agree<br>2. Agree<br>3. Not sure<br>4. Disagree<br>5. Strongly disagree
419	Does this disease affect your work performance?	1. Strongly agree<br>2. Agree<br>3. Not sure<br>4. Disagree<br>5. Strongly disagree
420	Does this disease affect marital relation?	1. Strongly agree<br>2. Agree<br>3. Not sure<br>4. Disagree<br>5. Strongly disagree
421	Does this disease affect family responsibilities?	1. Strongly agree<br>2. Agree<br>3. Not sure<br>4. Disagree<br>5. Strongly disagree

422	Does this disease affect your family relations?	1. Strongly agree<br>2. Agree<br>3. Not sure<br>4. Disagree<br>5. Strongly disagree

FOOD INSECURITY EXPERIENCE SCALE			
Now I would like to ask you some questions about your food consumption in the last 12 months. During the last 12 MONTHS, was there a time when:			
**S.N**	**Question**	**Response**	**Remark**
503	You were worried you would run out of food because of a lack of money or other resources?	1. Yes<br>2. No	
504	You were unable to eat healthy and nutritious food because of a lack of money or other resources?	1. Yes<br>2. No	
505	Did you eat only a few kinds of foods because of a lack of money or other resources?	1. Yes<br>2. No	
506	You had to skip a meal because there was not enough money or other resources to get food?	1. Yes<br>2. No	
507	You ate less than you thought you should because of a lack of money or other resources?	1. Yes<br>2. No	
508	Your household ran out of food because of a lack of money or other resources?	1. Yes<br>2. No	
509	You were hungry but did not eat because there was not enough money or other resources for food?	1. Yes<br>2. No	
510	Did you go without eating for a whole day because of a lack of money or other resources?	1. Yes<br>2. No	

Part VI: Behavioral Characteristics and other risk factors

601	Smoking status	1. Current<br>2. Former<br>3.Never
602	Alcohol drinking	1. Current<br>2. Former<br>3.Never
603	Co-existence of chronic diseases (other than HIV/AIDS)	1. Yes<br>2. No
