# Supplementary material for: Patient delay and associated factors among tuberculosis patients in Gamo zone public health facilities, Southern Ethiopia: An institution-based cross-sectional study
Source: PLoS One. 2021 Jul 30;16(7):e0255327. doi: 10.1371/journal.pone.0255327 (PMC8323940; doi:10.1371/journal.pone.0255327)
Supplement: Attachment — Submitted filename: Response to Editor Comments.docx [file pone.0255327.s005.docx]

**Response for editor’s comments**

**Dear Editors:**

We greatly appreciate your time dealing with the manuscript and assessing the review comments. The suggestions are quite helpful for us and we have made all necessary changes in our manuscript (**PONE-D-20-27080R1**) and incorporate them in the revised paper to address both editors and reviewer’s concern. From the changes made in the revised manuscript and responses provided below, I hope you are convinced that we have adequately addressed the reviewer’s concern and made the paper stronger. If there are any further questions, please feel free to let me know.

Thank you very much.

Sincerely,

On behalf of the co-authors

Asrat Arja, BSc, MPH

2021/04/4

**Authors:** Asrat Arja, Wanzahun Godana, Hadiya Hassen & Biruk Bogale

**Title**: Patient delay and associated factors among tuberculosis patients in Gamo zone public health facilities, Southern Ethiopia: An institution-based cross-sectional study

**Comment #1**: Please remove your figures/ from within your manuscript file, leaving only the individual TIFF/EPS image files. These will be automatically included in the reviewer’s PDF.

Authors ‘response: Thank you for pointing this out. We agree with this comment. Therefore, we have removed the figures from the manuscript and included the TFFS format in the supportive information's list with the respective figure number and short title.

**Comment #2:** Please ensure that you refer to Tables 3 and 5 in your text as, if accepted, production will need these references to link the reader to the Tables.

Authors ‘response: Thank you for your kindly review. It was also raised as comment in the previous reviewing process and accordingly we have made all the necessary correction and citations number in text are now in line with tables numbers.

**Comment #3:** Thank you for resubmitting your manuscript, we apologize for the delay in following up with you. We note some of the overlap within the discussion and conclusion section has not yet been addressed.

Authors ‘response: Again, thank you very much for your very kindly and careful review. Regarding the overlap within the discussion and conclusion section of the manuscript has now been comprehensively edited; including paraphrasing on the observed findings was done. We agree with this and have incorporated your suggestion throughout the manuscript.
